# Social Interactions as a Source of Information about E-Cigarettes: A Study of U.S. Adult Smokers

**DOI:** 10.3390/ijerph13080788

**Published:** 2016-08-05

**Authors:** Marissa G. Hall, Jessica K. Pepper, Jennifer C. Morgan, Noel T. Brewer

**Affiliations:** 1 Department of Health Behavior, Gillings School of Global Public Health, University of North Carolina, Rosenau Hall CB7440, Chapel Hill, NC 27599, USA; mghall@unc.edu (M.G.H.); jpepper@rti.org (J.K.P.); morganjc@email.unc.edu (J.C.M.); 2 Lineberger Comprehensive Cancer Center, University of North Carolina, Chapel Hill, NC 27599, USA; 3 RTI International, 3040 East Cornwallis Road, Research Triangle Park, NC 27709, USA

**Keywords:** social interactions, interpersonal communication, e-cigarettes, electronic nicotine delivery systems (ENDS), tobacco control

## Abstract

The novelty of e-cigarettes and ambiguity about their effects may foster informal sharing of information, such as through social interactions. We aimed to describe smokers’ social interactions about e-cigarettes and their recommendations that others use e-cigarettes. Data were collected from 2149 adult smokers in North Carolina and California who participated in a study of the impact of pictorial cigarette pack warnings. In the previous month, almost half of participants (45%) reported talking to at least one person about e-cigarettes and nearly a third of participants (27%) recommended e-cigarettes to someone else. Smokers recommended e-cigarettes to cut back on smoking (57%), to quit smoking (48%), for health reasons (36%), and for fun (27%). In adjusted analyses, more frequent e-cigarette use, positive views about typical e-cigarette users, and attempting to quit smoking in the past month were associated with recommending e-cigarettes for health reasons (all *p* < 0.05). Social interactions appear to be a popular method of information-sharing about e-cigarettes among smokers. Health communication campaigns may help to fill in the gaps of smokers’ understanding of e-cigarettes and their long-term effects.

## 1. Introduction

Use of electronic cigarettes (e-cigarettes) has increased rapidly in the U.S., particularly among smokers [1,2]. E-cigarettes likely pose less risk to individuals’ health compared to traditional cigarettes because they typically produce fewer harmful constituents [3]. However, the population-level risks and long-term health effects associated with widespread e-cigarette use are less clear. In particular, the extent to which e-cigarettes may facilitate smoking cessation remains a hotly-debated issue; two reviews have found that e-cigarettes may facilitate quitting smoking [4,5], whereas two reviews have concluded that e-cigarettes hinder smoking cessation [6,7].

Because of the novelty of the product and the lack of clear information about the effects of e-cigarettes, users and non-users may rely on informal methods of sharing information. Social interactions are a common method of information-sharing that are hypothesized to play a key role in shaping attitudes and behavior [8,9,10,11]. Smokers are more likely to socialize with other smokers [12], suggesting that social interactions could be an important mechanism for sharing information about e-cigarettes within this population. Indeed, two studies have demonstrated that adults frequently hear about e-cigarettes for the first time through word-of-mouth [13,14]. A nationally representative study found that word-of-mouth was the largest channel for sharing information about e-cigarettes, followed by Facebook and texting [15].

Past studies have examined social interactions about e-cigarettes, and these studies have largely focused on sources of awareness of and information about e-cigarettes. These studies have not explored the content of e-cigarette-related conversations, nor with whom smokers talk about e-cigarettes. Moreover, we do not know to what extent smokers actively recommend e-cigarettes to members of their social networks. A deeper examination of the nature and content of social interactions about e-cigarettes may shed light on how smokers develop opinions about e-cigarettes. To that end, we aimed to (1) describe the frequency and content of adult smokers’ conversations about e-cigarettes; (2) describe who smokers recommend e-cigarettes to and why they recommend them; and (3) identify factors that influence whether smokers recommend e-cigarettes for health reasons.

## 2. Materials and Methods

### 2.1. Participants

Participants were age 18 or older, proficient in English, and current smokers, defined as having smoked at least 100 cigarettes during their lifetime and now smoking every day or some days. Exclusion criteria included pregnancy, concurrent enrollment in a smoking cessation trial, smoking only roll-your-own cigarettes, smoking fewer than 7 cigarettes per week, and living in the same household as another study participant. We recruited participants in North Carolina and California, U.S. from September 2014 to August 2015 through Facebook, Craigslist, e-mail lists, in-person recruitment, referrals from local retailers, flyers, yard signs, bus advertisements, and newspaper advertisements.

### 2.2. Procedures

We conducted a randomized controlled trial comparing the impact of pictorial versus text-only warnings with adult smokers. Trial arm was not associated with our outcome of recommending e-cigarettes to others (*p* = 0.25). Details regarding recruitment, design, and methods appear in Brewer et al. [16]. Briefly, participants brought in an eight-day supply of cigarettes weekly for four weeks and were randomly assigned to have pictorial warnings applied to the top half of the front and back panels of their cigarettes packs or text-only warnings applied to the side of their cigarette packs. Participants completed weekly surveys on a computer at the study site and received a cash incentive at the end of each visit, up to a maximum of $185 in North Carolina and $200 in California. The baseline survey included the e-cigarette items used in the current study. At the end of the final follow-up appointment, participants received information about local smoking cessation programs. The University of North Carolina Institutional Review Board approved the study procedures (study number 13-2861).

### 2.3. Measures

We cognitively tested survey items with 10 adult smokers who had used an e-cigarette at least once in the past month [17]. At baseline, we assessed demographic characteristics and frequency of e-cigarette use. The baseline survey also asked about the nature of conversations about e-cigarettes, including the number of conversations about e-cigarettes in the past month, conversation partners, and topics of conversations. The survey also assessed views about typical e-cigarette users (i.e., prototypes [18,19,20,21]) by asking about participants’ opinion of the typical e-cigarette user. Responses ranged from very negative (1) to very positive (5). Finally, the survey assessed whether participants had ever recommended e-cigarettes to anyone else, to whom they recommended e-cigarettes, and why they recommended them. The survey items appear in the Supplementary Materials.

### 2.4. Data Analysis

Analyses used Stata/SE version 14.1 (StataCorp. LP, College Station, TX, USA) with two-tailed tests and a critical alpha of 0.05. First, we examined the prevalence of talking about e-cigarettes in the past month. Then, among those who had talked about e-cigarettes in the past month, we examined with whom participants talked about e-cigarettes and what they talked about. Next, among participants who had recommended e-cigarettes, we examined to whom people recommended e-cigarettes and why they recommended e-cigarettes. We also examined whether reasons for recommending e-cigarettes varied by type of social contact.

Finally, we examined correlates of recommending e-cigarettes for health reasons, which we dichotomized as yes (recommended e-cigarettes to quit smoking, to cut back on smoking, or for health reasons) or no. We excluded from these analyses 64 participants who recommended e-cigarettes only for fun. We conducted bivariate linear regressions to examine predictors of recommending e-cigarettes for health reasons. We then entered variables with *p*-values < 0.10 into a single multivariate model. Analyses used listwise deletion for missing data, excluding cases with incomplete data on the variables of interest from the model.

## 3. Results

From October 2014 to September 2015, we enrolled 2149 adult current smokers. Study participants were diverse, including a substantial number of African American, sexual minority, low-education, and low-income smokers (Table 1). About a third of participants (36%) had tried an e-cigarette but not in the past month, 11% had used an e-cigarette in the past month, and 15% had used an e-cigarette in the past week.

### 3.1. Conversations about E-Cigarettes

**Figure 1:**
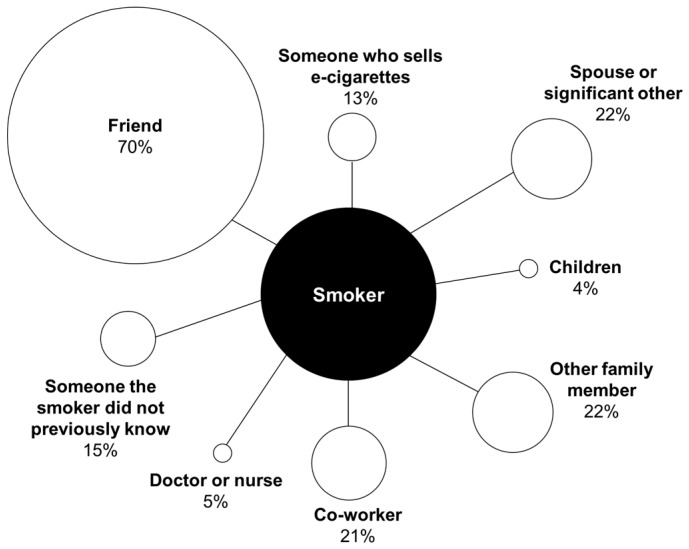
Conversation partners about e-cigarettes in past month (*n* = 962).

Nearly half of smokers (45%) reported talking to at least one person about e-cigarettes in the past month. These smokers talked to an average of 2.1 people about e-cigarettes during this time period. About two-thirds (69%) talked to an e-cigarette user, and 42% talked to a non-user. Among participants who talked to someone about e-cigarettes in the past month, most (70%) reported talking with a friend (Figure 1). Participants most commonly talked about using e-cigarettes to quit or cut back on smoking (58%; Table 2), what e-cigarettes are or how they work (43%), and preferences for brand, type, or flavor (41%).

### 3.2. Recommending E-Cigarettes

**Figure 2:**
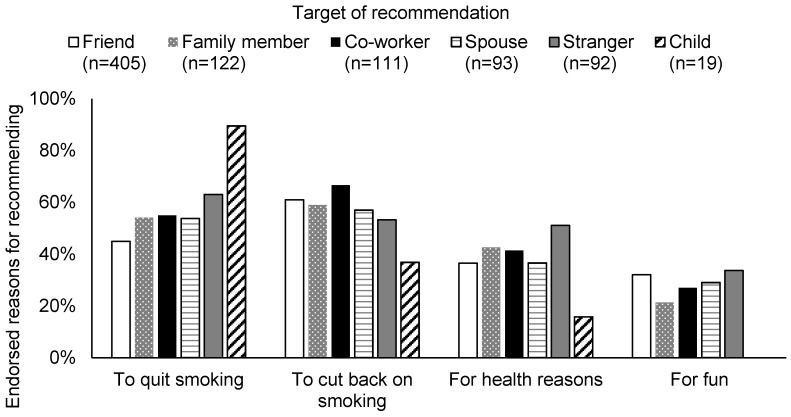
Reasons for recommending e-cigarettes to social contacts (*n* = 577).

Nearly a third of participants (577/2149, 27%) had recommended e-cigarettes to someone else (Table 1). Among these 577 people, the most common recommendation was to friends (71%), but it was also common to recommend to family members (21%), co-workers (19%), spouse or significant other (17%), someone they did not previously know (16%), and their children (3%). Participants recommended e-cigarettes for an average of 1.7 reasons, most commonly recommending them to cut back on smoking (57%), to quit smoking (48%), for health reasons (36%), and for fun (27%). The reasons for recommending e-cigarettes varied somewhat by type of social contact (Figure 2). For example, smokers frequently recommended that their children use e-cigarettes to quit smoking, but never for fun. Due to small cell sizes and the exploratory nature of these analyses, we did not test whether differences were statistically significant.

In adjusted analyses, each additional day of e-cigarette use in the past month was associated with 1.15 greater odds of recommending e-cigarettes for health reasons (*p* < 0.001; Table 3). Positive e-cigarette user prototypes were associated with greater odds of recommending e-cigarettes for health reasons (OR = 1.99, *p* < 0.001), as was having made a quit attempt in the past month (OR = 1.40, *p* < 0.05). Older participants and Black or African American participants had lower odds of recommending e-cigarettes for health reasons (OR = 0.97 and OR = 0.51, respectively, both *p* < 0.001). Variables associated in bivariate but not multivariate analyses were living in North Carolina; being gay, lesbian, or bisexual; Hispanic ethnicity; and having attended some college. The pattern of statistical significance for multivariate regression was identical when restricting analyses to smokers who had tried e-cigarettes.

## 4. Discussion

In this study, we aimed to describe the frequency and content of smokers’ conversations about e-cigarettes, finding that conversations about e-cigarettes were common in our study, with nearly half of participants talking to someone else about e-cigarettes in the past month. Smokers reported talking about e-cigarettes with their friends, family members (including children), co-workers, medical professionals, and even strangers. Conversations most commonly focused on using e-cigarettes to quit or cut back on smoking, what e-cigarettes are or how they work, and preferences for brand, type, or flavor. Two previous studies provide additional evidence that conversations about e-cigarettes are very prevalent. One study found that in a 2012 U.S. probability sample of 10,041 adults, in-person conversation was the second-most common source of hearing of e-cigarettes, after television [14]. Similarly, a 2013 national sample of 17,522 U.S. adults found that the most commonly-reported source of hearing of e-cigarettes was another person [13]. This study also found that word-of-mouth was the most common method of sharing information about e-cigarettes [15]. As awareness of e-cigarettes has increased to nearly universal levels [13], interpersonal communication may extend beyond merely introducing the concept of e-cigarettes to more substantive commentary about how e-cigarettes work and how they affect your health. Indeed, the conversations in our study focused on the health effects of e-cigarettes and whether they can help smokers quit or cut back on smoking. These findings suggest that informal conversations may be filling in the gaps that have resulted from a lack of understanding about the longer-term effects of e-cigarettes.

We also aimed to describe who smokers recommend e-cigarettes to and why they recommend them. Nearly a third of our sample of smokers had recommended e-cigarettes to someone, most commonly to friends, family members, co-workers, and spouses/significant others. Smokers recommended e-cigarettes to cut back on smoking, to quit smoking, for health reasons, and for fun. Although some evidence indicates that e-cigarettes may help people quit smoking [4,5], other studies suggest that e-cigarettes may make it harder to quit [6,7], potentially leading to dual use (i.e., concurrent use) of both cigarettes and e-cigarettes. Dual use of these products, even with the goal of reducing cigarette consumption, is concerning because quitting smoking altogether yields much greater health benefits than reducing smoking [22]. Moreover, gradual reduction in smoking is associated with lower rates of long-term abstinence compared to abrupt quitting [23]. In light of conflicting research on e-cigarettes as a cessation tool, the American Heart Association in the U.S. determined that there is not enough evidence for physicians to recommend e-cigarettes as a primary cessation aid, but that physicians should be prepared to counsel smokers on e-cigarettes and support their use in certain circumstances [24]. Smokers’ recommendations that others use e-cigarettes to quit or cut back on smoking should not take the place of physician counseling. The extent to which smokers’ recommendations promote e-cigarette use and dual use merits attention in future longitudinal studies.

Finally, we aimed to identify factors that influenced whether smokers recommend e-cigarettes for health reasons. We found that frequency of e-cigarette use was associated with greater odds of recommending e-cigarettes for health reasons. Frequent users may have positive experiences with and attitudes toward e-cigarettes, which could drive them to recommend use to others. We also found that smokers who had positive perceptions of the typical e-cigarette user (i.e., positive e-cigarette user prototypes) were more likely to recommend e-cigarettes for health reasons. Positive images of e-cigarettes and e-cigarette users prevail in tobacco industry advertising [25,26,27,28], which could lead to positive prototypes of e-cigarette users. These prototypes could not only increase willingness to use e-cigarettes as hypothesized in the prototypes/willingness model [20], but could also drive smokers to recommend e-cigarettes to others. Smokers who had made a quit attempt in the past month were more likely to recommend e-cigarettes for health reasons. As trying to quit or reduce smoking is a common reason for continued use of e-cigarettes among smokers [29], those who have attempted to quit smoking may subsequently be more likely to recommend using e-cigarettes for health reasons than other smokers.

The interaction between mass media campaigns and interpersonal communication merits careful attention in future research. Social interactions about e-cigarettes could be harmful if these interactions spread misinformation. Alternatively, social interactions can be a powerful tool for shaping norms, attitudes, and behavior [8,9,11], and thus could be harnessed to improve public health. Mass media campaigns and policy-level interventions such as warning labels have been shown to spark interpersonal communication. For example, an evaluation of a national anti-smoking campaign in the U.S., “Tips from Former Smokers”, found that the campaign resulted in millions more non-smokers talking about the dangers of smoking and recommending cessation services to friends or family [30]. Similarly, studies in Australia, the U.S., Canada, and Mexico have found that health warnings on cigarette packs trigger social interactions [10,31,32]. Conversations about e-cigarettes occurred frequently in our study, despite the lack of concurrent mass media campaigns or health warnings about e-cigarettes in the U.S. Social interactions about e-cigarettes in the U.S. may increase following the implementation of the new e-cigarette warning label required through the 2016 U.S. Food and Drug Administration deeming regulation [33]. Implementing well-designed health communication campaigns about e-cigarettes would likely elicit even more conversations about e-cigarettes and could shape the nature of these social interactions in order to influence attitudes and behavior about e-cigarettes. However, designing appropriate messages about e-cigarettes will be challenging given the dearth of research on the long-term effects of e-cigarettes.

Strengths of this study include a large sample of smokers and novel, cognitively-tested measures on social interactions about e-cigarettes. However, the generalizability of our results to other populations, such as non-smokers or rural smokers, has yet to be established. These data are largely exploratory, so we are unable to determine how social interactions may influence subsequent e-cigarette-related attitudes and behavior. Relying on self-report may have limited the accuracy of recalled information about social interactions. The data were collected in the context of a randomized controlled trial, but trial arm was not associated with our outcome of recommending e-cigarettes to others.

## 5. Conclusions

Our study of 2149 U.S. adult smokers described participants’ conversations about e-cigarettes (Aim 1), who they recommend e-cigarettes to and why they recommend them (Aim 2), and which factors influenced whether smokers recommend e-cigarettes for health reasons (Aim 3). We found that conversations about e-cigarettes were prevalent and most frequently focused on using e-cigarettes to quit or cut back on smoking. Nearly a third of smokers recommended e-cigarettes to others, usually for health reasons such as quitting or cutting back on smoking. Frequency of e-cigarette use, positive e-cigarette user prototypes, and making a quit attempt were associated with a greater likelihood of recommending e-cigarettes for health reasons. Conversations about e-cigarettes are a popular source of information-sharing among smokers, highlighting an opportunity for health communication campaigns to shape the nature of these conversations. Future research should examine the extent to which social interactions influence subsequent attitudes, norms, and behaviors about e-cigarettes.

## Figures and Tables

**Table 1:** Participant characteristics at baseline (*n* = 2149).

Characteristic	*n*	(%)
Age		
18–24 years	323	(15.3)
25–39 years	775	(36.7)
40–54 years	642	(30.4)
55+ years	371	(17.6)
Mean (SD)	39.7	(13.5)
Gender		
Male	1039	(48.7)
Female	1060	(49.7)
Transgender	34	(1.6)
Gay, lesbian, or bisexual	368	(17.5)
Hispanic	181	(8.6)
Race		
White	751	(35.7)
Black or African American	994	(47.3)
American Indian or Alaska Native	18	(0.9)
Asian	70	(3.3)
Native Hawaiian or other Pacific Islander	17	(0.8)
Other/multiracial	251	(12.0)
Education		
High school degree or less	677	(31.8)
Some college	1021	(47.9)
College graduate	312	(14.6)
Graduate degree	121	(5.7)
Low income (≤150% of Federal Poverty Level)		
No	983	(45.9)
Yes	1159	(54.1)
Household income, annual		
$0–$24,999	1155	(54.5)
$25,000–$49,999	538	(25.4)
$50,000–$74,999	202	(9.5)
$75,000+	224	(10.6)
Study site		
California	1186	(55.2)
North Carolina	963	(44.8)
Cigarettes smoked per day, mean (SD)	8.8	(6.9)
Made quit attempt in past month	545	(26.5)
E-cigarette use		
Never used	828	(39.0)
Used but not in past month	750	(35.5)
Used in past month	223	(10.6)
Used in past week	312	(14.8)
Number of times used e-cigarette in past month, mean (SD)	1.38	(4.1)
Talked to someone about e-cigarettes in past month	962	(45.3)
Recommended e-cigarettes for any reason	577	(27.2)
Recommended e-cigarettes for health reasons	513	(24.2)

Missing demographic data range from 0.7% to 2.2%.

**Table 2:** Topics of conversation about e-cigarettes (*n* = 962).

Topic	*n* (%)
Using them to quit or cut back on smoking	557 (58)
What e-cigarettes are or how they work	412 (43)
Preferences for brand, type, or flavor	393 (41)
Where to buy them or how much they cost	365 (38)
How they affect your health	293 (30)
Where I can use them	229 (24)

**Table 3:** Correlates of recommending e-cigarettes for health reasons (*n* = 1820).

Variable	Bivariate OR	Multivariate OR
Number of times used e-cigarette in past month	1.20 **	1.15 **
Positive e-cigarette user prototypes	1.97 **	1.99 **
Made quit attempt in past month		
No (ref)	-	-
Yes	1.45 **	1.40 **
Study site		
California (ref)	-	-
North Carolina	1.28 **	1.13**
Age	0.96 **	0.97 **
Gender		
Male (ref)	-	-
Female	0.92**	-
Transgender	0.85**	-
Sexual orientation		
Straight (ref)	-	-
Gay, lesbian, or bisexual	1.45 **	1.16**
Hispanic ethnicity		
Not Hispanic (ref)	-	-
Hispanic	1.75 **	1.32**
Race		
White (ref)	-	-
Black or African American	0.46 **	0.51 **
Other	0.94**	0.78**
Education		
High school degree or less (ref)	-	-
Some college	1.38 **	1.19**
College graduate	1.01**	0.79**
Low income (<150% of FPL)		
No (ref)	-	-
Yes	1.03**	-
Number of cigarettes smoked per day	1.01**	-

Analyses included data from 1820 smokers, a sample that reflects the exclusion of 64 participants who recommended e-cigarettes only for fun and 265 additional participants with missing values for at least one of the predictor variables. The multivariate model included variables with *p*-values < 0.10 in bivariate analyses. * *p* < 0.05, ** *p* < 0.001.

## Supplementary Materials

The following are available online at www.mdpi.com/1660-4601/13/8/788/s1, Table S1: Survey items.
