# Supplementary material for: Social Interactions as a Source of Information about E-Cigarettes: A Study of U.S. Adult Smokers
Source: Int J Environ Res Public Health. 2016 Aug 5;13(8):788. doi: 10.3390/ijerph13080788 (PMC4997474; doi:10.3390/ijerph13080788)
Supplement: Supplementary file 1 [file ijerph-13-00788-s001.pdf]

Supplementary Materials: Social Interactions as a Source of Information about E-Cigarettes: A Study of U.S. Adult Smokers

Marissa G. Hall, Jessica K. Pepper, Jennifer C. Morgan and Noel T. Brewer

Table S1. Survey items.

Construct	Item	Response Scale
Ever used e-cigarette	Have you ever used an e-cigarette or other vaping device, even one or two times?	0 = No<br>1 = Yes
Past month e-cigarette use	In the last 4 weeks, on how many days did you use an e-cigarette or other vaping device?	Number of days
Past week e-cigarette use	In the last week, on how many days did you use an e-cigarette or other vaping device?	Number of days
Frequency of conversations about e-cigarettes	In the last month, how many people did you talk to about e-cigarettes?	1 = No one<br>2 = 1–2 people<br>3 = 3–4 people<br>4 = 5 or more people
Conversation partners	In the last month, who did you talk to about e-cigarettes?—My spouse or significant other	0 = No<br>1 = Yes
Conversation partners	In the last month, who did you talk to about e-cigarettes—My child	0 = No<br>1 = Yes
Conversation partners	In the last month, who did you talk to about e-cigarettes—Other family member	0 = No<br>1 = Yes
Conversation partners	In the last month, who did you talk to about e-cigarettes—Friend	0 = No<br>1 = Yes
Conversation partners	In the last month, who did you talk to about e-cigarettes—Co-worker	0 = No<br>1 = Yes
Conversation partners	In the last month, who did you talk to about e-cigarettes—Doctor or nurse	0 = No<br>1 = Yes
Conversation partners	In the last month, who did you talk to about e-cigarettes—Someone who sells e-cigarettes	0 = No<br>1 = Yes
Conversation partners	In the last month, who did you talk to about e-cigarettes—A stranger	0 = No<br>1 = Yes
Conversation topics	In the last month, when discussing e-cigarettes, what did you talk about—What e-cigarettes are or how they work	0 = No<br>1 = Yes
Conversation topics	In the last month, when discussing e-cigarettes, what did you talk about—Using them to quit or cut back on smoking	0 = No<br>1 = Yes
Conversation topics	In the last month, when discussing e-cigarettes, what did you talk about—Where to buy them or how much they cost	0 = No<br>1 = Yes
Conversation topics	In the last month, when discussing e-cigarettes, what did you talk about—Preferences for brand, type, or flavor	0 = No<br>1 = Yes
Conversation topics	In the last month, when discussing e-cigarettes, what did you talk about—How they affect your health	0 = No<br>1 = Yes
Conversation topics	In the last month, when discussing e-cigarettes, what did you talk about—Where I can use them	0 = No<br>1 = Yes
Ever recommended e-cigarette	Have you ever recommended that someone use e-cigarettes?	0 = No<br>1 = Yes
Who recommended to	Who did you recommend e-cigarettes to?—My spouse or significant other	0 = No<br>1 = Yes
Who recommended to	Who did you recommend e-cigarettes to?—My child	0 = No<br>1 = Yes

Who recommended to	Who did you recommend e-cigarettes to?—Other family member	0 = No<br>1 = Yes
Who recommended to	Who did you recommend e-cigarettes to?—Friend	0 = No<br>1 = Yes
Who recommended to	Who did you recommend e-cigarettes to?—Co-worker	0 = No<br>1 = Yes
Who recommended to	Who did you recommend e-cigarettes to?—A stranger	0 = No<br>1 = Yes
Why recommended	Why did you recommend e-cigarettes to your spouse or significant other—To quit smoking	0 = No<br>1 = Yes
Why recommended	Why did you recommend e-cigarettes to your spouse or significant other—To cut back on smoking	0 = No<br>1 = Yes
Why recommended	Why did you recommend e-cigarettes to your spouse or significant other—For health reasons	0 = No<br>1 = Yes
Why recommended	Why did you recommend e-cigarettes to your spouse or significant other—For fun	0 = No<br>1 = Yes
Why recommended	Why did you recommend e-cigarettes to your child?—To quit smoking	0 = No<br>1 = Yes
Why recommended	Why did you recommend e-cigarettes to your child?—To cut back on smoking	0 = No<br>1 = Yes
Why recommended	Why did you recommend e-cigarettes to your child?—For health reasons	0 = No<br>1 = Yes
Why recommended	Why did you recommend e-cigarettes to your child?—For fun	0 = No<br>1 = Yes
Why recommended	Why did you recommend e-cigarettes to another family member—To quit smoking	0 = No<br>1 = Yes
Why recommended	Why did you recommend e-cigarettes to another family member—To cut back on smoking	0 = No<br>1 = Yes
Why recommended	Why did you recommend e-cigarettes to another family member?—For health reasons	0 = No<br>1 = Yes
Why recommended	Why did you recommend e-cigarettes to another family member?—For fun	0 = No<br>1 = Yes
Why recommended	Why did you recommend e-cigarettes to a friend?—To quit smoking	0 = No<br>1 = Yes
Why recommended	Why did you recommend e-cigarettes to a friend?—To cut back on smoking	0 = No<br>1 = Yes
Why recommended	Why did you recommend e-cigarettes to a friend?—For health reasons	0 = No<br>1 = Yes
Why recommended	Why did you recommend e-cigarettes to a friend?—For fun	0 = No<br>1 = Yes
Why recommended	Why did you recommend e-cigarettes to a co-worker—To quit smoking	0 = No<br>1 = Yes
Why recommended	Why did you recommend e-cigarettes to a co-worker—To cut back on smoking	0 = No<br>1 = Yes
Why recommended	Why did you recommend e-cigarettes to a co-worker—For health reasons	0 = No<br>1 = Yes
Why recommended	Why did you recommend e-cigarettes to a co-worker—For fun	0 = No<br>1 = Yes
Why recommended	Why did you recommend e-cigarettes to a stranger?—To quit smoking	0 = No<br>1 = Yes
Why recommended	Why did you recommend e-cigarettes to a stranger?—To cut back on smoking	0 = No<br>1 = Yes
Why recommended	Why did you recommend e-cigarettes to a stranger?—For health reasons	0 = No<br>1 = Yes
Why recommended	Why did you recommend e-cigarettes to a stranger?—For fun	0 = No<br>1 = Yes

E-cigarette prototypes	Picture a typical e-cigarette user your age. Is your opinion of this person…?	1 = Very negative<br>2 = Somewhat negative<br>3 = Neutral<br>4 = Somewhat positive<br>5 = Very positive
Past month quit attempt	During the last 4 weeks, did you stop smoking for 1 day or longer because you were trying to quit smoking?	0 = No<br>1 = Yes

© 2016 by the authors; licensee MDPI, Basel, Switzerland. This article is an open access article distributed under the terms and conditions of the Creative Commons by Attribution (CC-BY) license (http://creativecommons.org/licenses/by/4.0/).
